# An Efficient Lightweight SAR Ship Target Detection Network with Improved Regression Loss Function and Enhanced Feature Information Expression

**DOI:** 10.3390/s22093447

**Published:** 2022-04-30

**Authors:** Jimin Yu, Tao Wu, Xin Zhang, Wei Zhang

**Affiliations:** College of Automation, Chongqing University of Posts and Telecommunications, Chongqing 400065, China; yujm@cqupt.edu.cn (J.Y.); S200331117@stu.cqupt.edu.cn (X.Z.); S190301072@stu.cqupt.edu.cn (W.Z.)

**Keywords:** lightweight network, regression loss function, SCUPA module, GCHE module, SAR ship detection

## Abstract

It is difficult to identify the ship images obtained by a synthetic aperture radar (SAR) due to the influence of dense ships, complex background and small target size, so a deep learning-based target detection algorithm was introduced to obtain better detection performance. However, in order to achieve excellent performance, most of the current target detection algorithms focus on building deep and high-width neural networks, resulting in bloated network structure and reduced detection speed, which is not conducive to the practical application of target detection algorithms. Thereby, an efficient lightweight network Efficient-YOLO for ship detection in complex situations is proposed in the present work. Firstly, a new regression loss function ECIOU is proposed to enhance the detection boxes localization accuracy and model convergence speed. Secondly, We propose the SCUPA module to enhance the multiplexing of picture feature information and the model generalization performance. Thirdly, The GCHE module is proposed to strengthen the network’s ability to extract feature information. At last, the effectiveness of our method is tested on the specialized ship dataset: SSDD and HRSID datasets. The results show that Efficient-YOLO outperforms other state-of-the-art algorithms in accuracy, recall and detection speed, with smaller model complexity and model size.

## 1. Introduction

Accurate object detection has great scientific and practical significance in ocean, forestry and traffic navigation, and has always been a research hotspot in the field of remote sensing. However, remote sensing images obtained from satellite sensors have different points of view compared with images directly acquired in real life (images directly observed or photos taken by humans). These remote sensing images contain diverse complex landscapes and are often more susceptible to atmospheric, background clutter, light differences and have less spatial details [1], which makes it a challenge to detect and identify objects in high-resolution remote sensing images timely and accurately. With the rapid development and innovation of wireless communication technology, sensor technology and other related disciplines, synthetic aperture radar (SAR) has emerged [2]. SAR is an active high-resolution microwave imaging sensor based on the principle of synthetic aperture. Its imaging process is less affected by environmental factors and can provide massive space-to-earth observation data under 24-h all-weather conditions.Therefore, SAR-based ship detection systems are widely used in maritime surveillance activities and play an increasingly important role.

In SAR image ship target detection, the Constant False Alarm Rate (CFAR) [3] algorithm is the most widely used in traditional methods. It detects ship targets by modeling the statistical distribution of background clutter. However, this type of algorithm has the problem of poor adaptability, which limits the application of migration, and the change of background often has a great influence on the detection result. In recent years, with the vigorous development of deep learning, the target detection technology based on the convolutional neural network has become more and more mature, and its accuracy and speed have been greatly improved. It gradually replaces the traditional method and is widely used in the field of ship inspection [4,5,6,7].

Generally speaking, the existing target detection methods can be divided into two-stage detection and single-stage detection. The two-stage detection algorithm divides the detection process into two stages. Firstly, the pre-selected boxes are generated by the region candidate network, and secondly, the classification and regression of the pre-selected boxes are realized by the detection network. Therefore, the algorithm has good positioning and target recognition accuracy, such as the R-CNN series [8,9,10]. However, the two-stage algorithm causes more occupation of computational resources due to the deeper network structure, with a slow detection speed and is difficult to use in the environments with real-time detection requirements. Single-stage detection algorithm directly obtains detection results through the detection network, thus it has high inference speed and is conducive to application on mobile devices with high real-time requirements, such as YOLO series [11,12,13,14], Retinanet [15] and SSD [16]. The YOLO series, as a representative method for single-stage detection, growing from YOLOv1 [11], YOLOv2 [12], YOLOv3 [13] and then YOLOv4 [14] proposed in 2020 further improved the detection performance. The CSPDarknet53 network proposed in YOLOv4 was a typical lightweight framework. This network was used as the backbone network for feature extraction, which enabled YOLOv4 to achieve a good balance between detection speed and accuracy in practical applications.

Inspired by these advanced methods, many remote sensing experts have tried to introduce deep learning into the field of SAR target detection [17,18]. In large-field SAR images, ships belong to the small target category, occupy fewer pixels and are highly vulnerable to background factors. However, since the unique imaging mechanism of the SAR image background region is much more complex than the optical image, higher requirements for the accuracy of the detection network are imposed. To pursue better detection accuracy, some scholars focus on building deep and high-width neural networks [19]. In this way, the computing complexity and model parameters of the neural network will be greatly improved, which will bring a huge load to small devices with limited computing resources, and will easily cause adverse effects such as system collapse and increased power consumption, which will bring great trouble to the actual application of target detection technology.

The detection accuracy and detection speed seem to be a pair of natural contradictions. Greatly improving the detection accuracy will bring the network structure of the deep learning-based target detection algorithm bloated, resulting in low real-time and extending the training time. However, in the pursuit of efficient speed, it is necessary to lightweight the network and streamlines the network structure, but this may lead to the network structure being too thin to fully extract the feature information and location information of the target to be detected, which will greatly reduce the detection performance of the network. For example, YOLOX-M has a simplified network structure and a small model size, but its detection performance is inferior to that of YOLOv4, which has a similar model architecture [20]. YOLOv4-Tiny obtains a faster detection speed through a large number of deletions of each model of the YOLOv4 network, but its detection ability is not ideal [21]. The above lightweight models are all at the expense of a certain detection performance to achieve rapid detection results through concise models. However, speed and accuracy can be jointly improved with effective improvement methods and reasonable model architecture, and it is not necessary to sacrifice detection accuracy to improve speed, while simplifying the network model, we can add effective modules that do not significantly increase the complexity of the network, design a reasonable architecture and optimize the loss function outside the network model to obtain an ideal detection model.

Therefore, in order to verify the above ideas, we propose an efficient and lightweight object detection algorithm Efficient-YOLO for ship detection in complex situations in this paper. Compared to other state-of-the-art methods, the detection framework proposed in this paper has less parameter amount and computational complexity and higher detection speed, while maintaining satisfactory accuracy. The main contributions of our work are summarized as follows.

1. A new regression loss function Efficient Complete Intersection over Union (ECIoU) is proposed to improve network performance without increasing network complexity. Its formula complexity is low, the effect is robust, the convergence speed is fast, the detection frame positioning accuracy is high, and the practical application performance is excellent.

2. Combined with Shuffle Depthwise separable convolution Based on ReLU6 (SDBR) module and Upsample-S module, a new feature fusion network SDBR-based style Channel Upsampling Path Aggregation (SCUPA) module is proposed. To put it simply, firstly, the SDBR convolution block is used to interactively fuse all channel information to enhance the reuse of image feature information and effectively improve the generalization performance of the model. Secondly, a new upsampling module (Upsample-S) is applied to further avoid the loss of image feature information caused by traditional upsampling operations.

3. A Gate Channel Head Embedded (GCHE) module is proposed to reduce the interference information in the network structure and enhance the ability of the network prediction part to extract feature information.

## 2. Related methods

### 2.1. Style-Based Recalibration (SRM) Module

**Figure 1:**
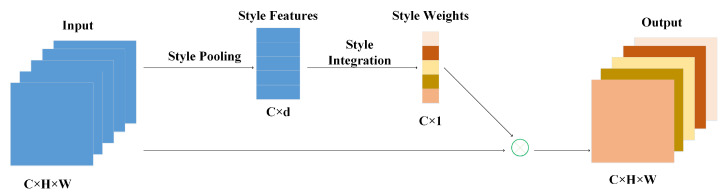
SRM network structure.

The SRM [22] is a simple and efficient architecture unit that adaptively recalibrates feature information through the style of intermediate feature maps. As shown in Figure 1. It is composed of two main components: Style Pooling and Style Integration. Style Pooling extracts style features from each channel by aggregating feature responses across spatial dimensions. First, given the input feature graph $X\in R^{N\times C\times H\times W}$ (where *N* indicates the number of examples in the mini-batch, *C* is the number of channels, *H* and *W* indicate spatial dimensions [22]), we obtain the channel-level statistics of the feature, namely the mean ($\mu_{nc}$) and the standard deviation ($\sigma_{nc}$), and finally obtain the feature graph style information $t_{nc}=\left[\mu_{nc},\sigma_{nc}\right]$. Then, $t_{nc}$ is multiplied by a learnable parameter $w_{c}$ for style integration to obtain the new style feature information $z_{nc}$, and the channel normalization and Sigmoid function are applied to process the feature information(where n and c indicate each example n and channel c, respectively). The specific process can be described as follows.

(1) $$\mu_{c}^{\left(z\right)}=\frac{1}{N}\sum_{n=1}^{N}z_{nc},$$

(2) $$\sigma_{c}^{\left(z\right)}=\sqrt{\frac{1}{N}\sum_{n=1}^{N}{\left(z_{nc}-\mu_{c}^{\left(z\right)}\right)}^{2}},$$

(3) $${\hat{z}}_{nc}=\gamma_{c}*\frac{z_{n}c-\mu_{c}^{\left(z\right)}}{\sigma_{c}^{\left(z\right)}}+\beta_{c},$$

(4) $$g_{nc}=\frac{1}{1+e^{-{\hat{z}}_{nc}}},$$

where $\gamma_{c}$ and $\beta_{c}$ are the affine transformation parameters, *N* represents the number of samples, *C* represents the number of channels, and $g_{nc}$ isthe final weight coefficient.

(5) $${\hat{x}}_{nc}=g_{nc}\cdot x_{nc},$$

where ${\hat{x}}_{nc}$ is the feature map information after self-calibration by SRM.

### 2.2. Gated Channel Transformation (GCT)

The gating mechanism has been successfully applied in several recurrent neural network structures. The Long Short Term Memory Network (LSTM) [23] introduced input, output, and forgetting gates to regulate the flow of information in and out of the module [24].

**Figure 2:**
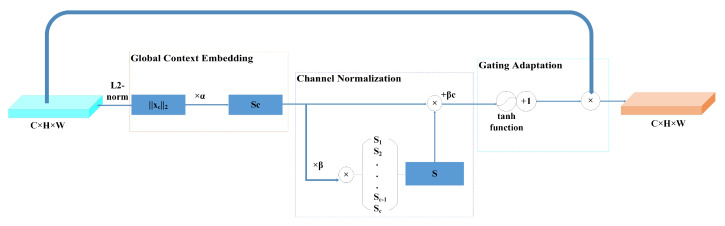
GCT network structure.

GCT [24] is a simple and effective architecture for modeling interchannel relationships which significantly improved the generalization power of deep convolutional networks on visual recognition tasks and datasets, as shown in Figure 2. GCT mainly consists of three partial—global context embeddings, channel normalization, and gating adaptation.

Firstly, the $l_{2}-norm$ is used to extract the global context information for each channel, defined as follows.

(6) $$s_{c}=\alpha_{c}\left|\right|x_{c}{\left|\right|}_{2}=\alpha_{c}{\left\{\left[\sum_{i=1}^{H}\sum_{j=1}^{W}{\left(x_{c}^{i,j}\right)}^{2}\right]+\epsilon \right\}}^{\left(1/2\right)},$$

where $\alpha_{c}$ is a trainable parameter to control the importance of different channels, $x_{c}$ is the input feature information and ϵ is minima.

Secondly, the channel normalization obtained by $s_{c}$ is ${\hat{s}}_{c}$.

(7) $${\hat{s}}_{c}=\frac{\sqrt{C}s_{c}}{\left|\right|s_{c}{\left|\right|}_{2}}=\frac{\sqrt{C}s_{c}}{{\left(\sum_{c=1}^{C}s_{c}^{2}+\epsilon \right)}^{1/2}},$$

where $\sqrt{C}$ is the scale factor.

Finally, the obtained information is input into the tanh function to obtain the final output through gating adaptation.

(8) $${\hat{x}}_{c}=x_{c}\left[1+tanh\left(\gamma_{c}{\hat{s}}_{c}+\beta_{c}\right)\right],$$

where $\gamma_{c}$ and $\beta_{c}$ are trainable parameters, they are used to control the channel threshold.

### 2.3. Depthwise Separable Convolution (DSC)

**Figure 3:**
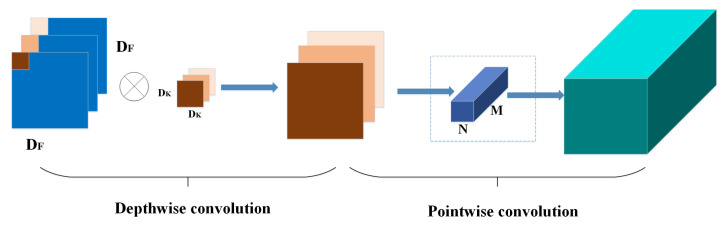
Depthwise separable convolution.

Depthwise separable convolution [25] is a convolution method proposed by MobileNetv1, which effectively improves the computational efficiency and reduced the number of convolutional networks compared to standardized convolution, and its ratio to traditional convolutional parameters is shown in Equation (1). In the operation of standardized convolution, each convolutional kernel has the corresponding parameters for each channel of the input feature graph, performing separate convolution operations, and then be added up to obtain the output feature graph. However, deep separable convolution is a decomposition of the standardized convolution into deep convolution and 1 × 1 convolution called point-wise convolution. In deep convolution, a single filter was applied to each input channel, and then, point by point, convolution with 1 × 1 to combine the output of different deep convolutions. The principle of deep separable convolution is shown in Figure 3.

(9) $$\frac{D_{K}\cdot D_{K}\cdot M\cdot D_{F}\cdot D_{F}+M\cdot N\cdot D_{F}\cdot D_{F}}{D_{K}\cdot D_{K}\cdot M\cdot N\cdot D_{F}\cdot D_{F}}=\frac{1}{N}+\frac{1}{D_{K}^{2}},$$

where $D_{K}\cdot D_{K}$ indicates the convolutional kernel size. *M* and *N* represent the input and output channels, respectively. $D_{F}\cdot D_{F}$ is the size of the feature graph. It can be seen that the parameters and computational cost of the network are significantly reduced.

## 3. Proposed Methods and Model Architecture

### 3.1. Efficient-YOLO Network

Although the current deep learning target detection algorithms have good performance, most of them have large size and computation, which is not conducive to the practical application of object detection algorithms.

**Figure 4:**
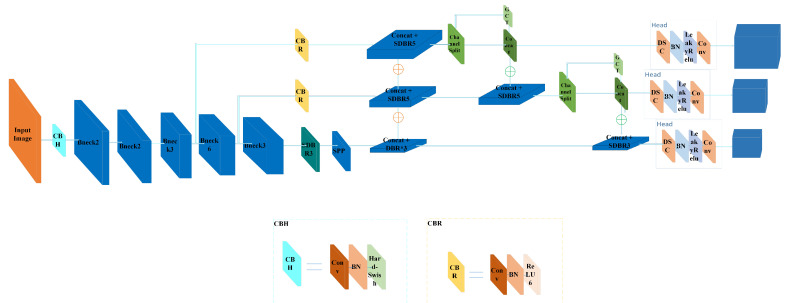
Efficient-YOLO network structure diagram.

To further obtain higher accuracy and smaller computation, the backbone network of this paper is built according to a reasonable architecture based on the MobileNetv3 basic modules, where BneckX represents X MobileNetv3 basic modules, while choosing P3, P4 and P5 as three different feature layers. The SPP module and the SCUPA module proposed in the present paper are selected as the neck network to realize the fusion of low-resolution and semantic strong features with more efficient high-resolution features. The GCHE module proposed in this paper is chosen as the prediction network, the ECIoU loss function serves as the regression loss function, and the cross entropy loss function is used to detect the confidence error and classification error. To significantly reduce the network parameters while maintaining the network detection performance without great loss, all 3 × 3 convolutions in the Efficient-YOLO proposed here employ deeply separable convolution. Due to the superiority of the YOLOv4 network architecture, the network model proposed in this paper basically follows its architecture. Determine experimentally that the proposed Efficient-YOLO network model can greatly reduce the number of parameters, and achieve higher accuracy and faster detection speed. As shown in Figure 4.

### 3.2. Backbone Network

#### MobileNetv3 Basic Module Structure

Currently, an increasing number of lightweight CNNs can significantly reduce the number of parameters and reduce the demand for computational resources, and gradually become the mainstream network in the field of deep learning.

**Figure 5:**
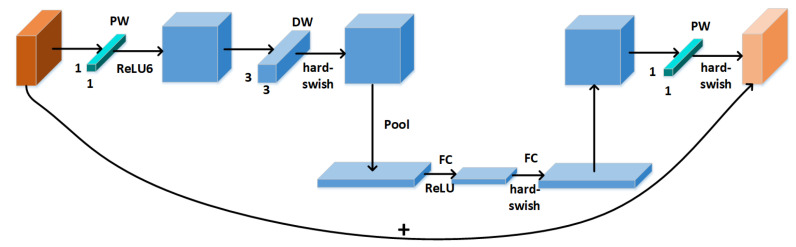
MobileNetv3 basic module.

MobileNetv3 network [26] is a lightweight deep neural network model. MobileNetv3 combined the deeply separable convolution of MobileNetv1 [25] and the inverse residual structure of MobileNetv2 [27]. Firstly, the high-dimensional feature information is preserved through three-layer convolution to reduce the delay of backpropagation. Secondly, the attention mechanism Squeeze-and-Excitation (SE) module [28] was introduced to weight each channel to improve the detection accuracy. The basic modules in the MobileNetv3 network are shown in Figure 5.

### 3.3. Neck Network

The neck network consists mainly of SCUPA, Spatial Pyramid Pooling (SPP) [29], an SDBR3 module and two 1 × 1 convolutions.

#### 3.3.1. Shuffle Depthwise Separable Convolution Based on ReLU6 (SDBR) Module

**Figure 6:**
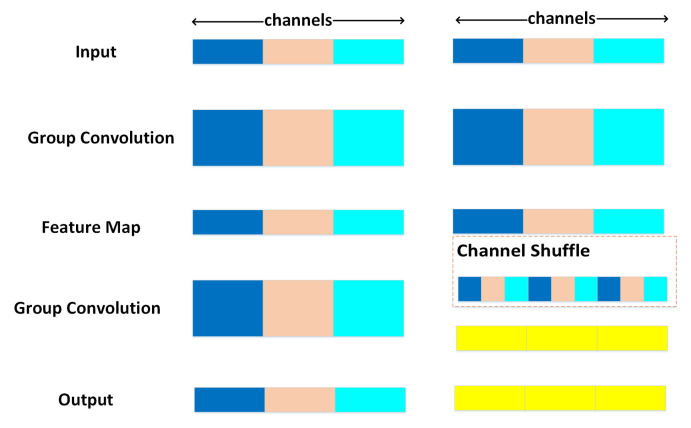
Schematic diagram of the Channel Shuffle process.

The channel shuffle mechanism [30] reorganizes the multi-channel feature map information after grouping convolution to ensure that the next network input feature information comes from different channels and transfers the information between each channel. This operation can effectively improve the generalization of the model. Specifically, this operation randomly divides the input channel of the model into two parts, one part retains its mapping and is directly passed down, the other part is calculated directly backward, and the part used here is half of the original input channel. At the bottom of the module, element-wise addition is avoided by cascading the output channels of the two branches. Then, random channel mixing is performed on the output feature map, and the feature information covering all channels is output downward. The model structure is shown in Figure 6.

**Figure 7:**
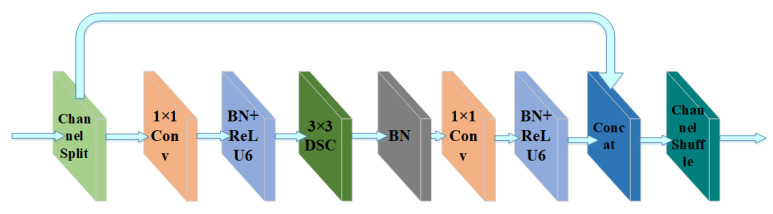
Network structure diagram of SDBR3.

**Figure 8:**
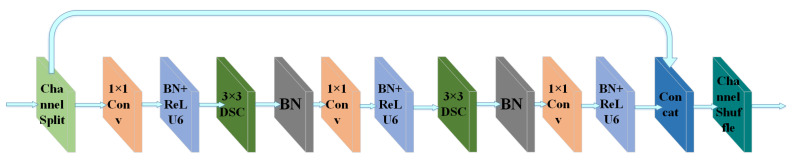
Network structure diagram of SDBR5.

**Figure 9:**
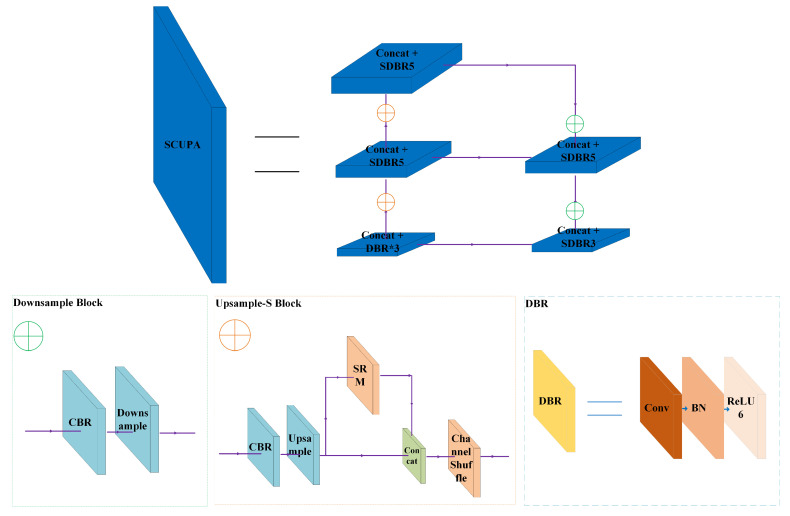
The SCUPA module structure.

Based on the cubic and quintic convolution blocks in Path Aggregation Network (PANet) [31], we propose new cubic and quintic convolution blocks based on the random channel shuffle mechanism in the present work, called SDBR3 and SDBR5 modules, respectively. SDBR3 is mainly composed of channel shuffle, depthwise separable convolution, 1 × 1 convolution, batch normalization and activation function ReLU6, where 3 represents the number of convolution layers. The composition of SDBR5 module is similar to SDBR3, but the number of internal operation layers of the module is more than that of SDBR3, as shown in Figure 7 and Figure 8. In this paper, the above two modules are, respectively, applied to the SCUPA module to extract feature information, as shown in Figure 9. This method can greatly reduce the network parameters of the feature fusion network, while enhancing the integrity of image feature information fusion and the effectiveness of feature information extraction.

#### 3.3.2. SDBR-Based Style Channel Upsampling Path Aggregation (SCUPA) Module

The path aggregation network (PANet) structure adds a top-down structure to the structure of the feature pyramid network (FPN) [32], which improves the original single bottom-up feature fusion and adds a top-down feature fusion line. It obtains larger scale and higher resolution fused feature layers. Different features are aggregated from different layers of the backbone network, and high-level semantic information and low-level information are fused, which further enhances the feature fusion capability of the network, and specifically improves the sensitivity of the detection algorithm to small target objects [33].

However, PANet has two disadvantages, affecting the fusion of network feature information. Firstly, a large number of five convolution blocks are directly used to extract the picture feature information. Although the detection effect is good, it does not make full use of the feature information, and the network model parameters are too large. Secondly, PANet uses the nearest proximity interpolation method to upsample the extracted feature map, resulting in the loss of the feature map information, making it difficult for the network to distinguish the main information of the feature map, and will blur the expression of the target feature information to be detected.

To address the above problems, the SCUPA module is presented in this paper, as shown in Figure 9. Firstly, we apply the SDBR5 module to replace all quintic convolution blocks in PANet, and replace one of the quintic convolution blocks with SDBR3 block. This method can effectively reduce the computational complexity of the network, improve the generalization performance of the model, and strengthen the reuse of image feature information. Secondly, in order to further reduce the impact of the upsampling operation on the expression of image information, we propose a new upsampling (Upsample-S) module by using the channel shuffle mechanism and the SRM module, which can further avoid the loss of image feature information caused by the upsampling operation.

#### 3.3.3. Spatial Pyramid Pooling (SPP) Module

In 2015, the SPP module was proposed to convert feature maps of any size into fixed-size eigenvectors. The SPP module can reduce the loss and deformation of the image feature information, and enhance the recognition accuracy of the network.

**Figure 10:**
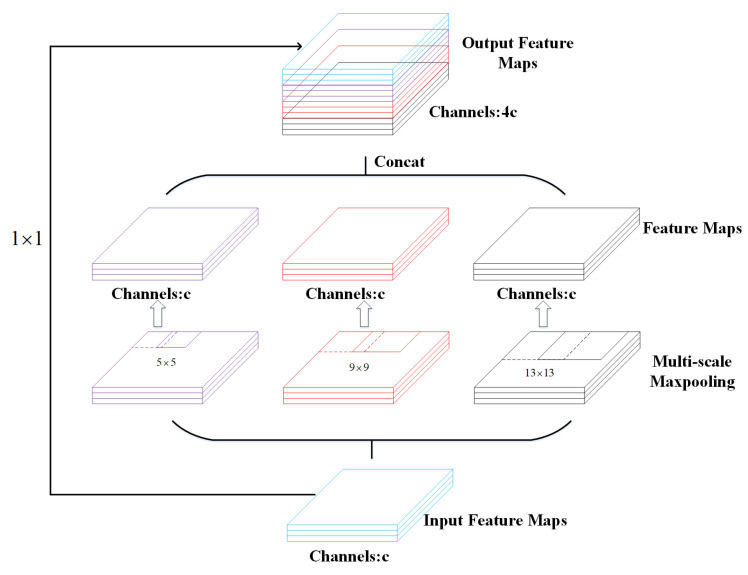
SPP module structure diagram.

In this paper, the Spatial Pyramid Pooling (SPP) is applied to the last layer of the Mobilenetv3 backbone extraction network to pool the output features and produce a fixed-length output. It can slice the image into coarse levels of fine and then integrate local features, thus greatly increasing the receptive field and separating the most prominent contextual features. As shown in Figure 10.

### 3.4. Prediction Network—Gate Channel Head Embedded (GCHE) Module

YOLOv4 follows the method of multiscale prediction, outputting three feature maps of different sizes to detect three target objects of large, medium and small, respectively. The detection head consists of two convolutional layers, with the final prediction obtained after using 3 × 3 and 1 × 1 convolutional layers [34]. The number of output channels for each final prediction is 3 (K + 5), which represents the prior box of the three sizes set by each layer where K is the number of categories. Five can be divided into 4 + 1, representing the parameter information of the central point coordinates (x, y) of the predicted bounding box, the width and height size (w, h) of the box, and the confidence, respectively.

However, when YOLOv4 uses the convolutional features extracted by the network prediction part, it does not weigh the different positions and channels in the convolutional kernel. That is, we treat each region in the whole feature graph equally, considering that each region has the same contribution to the final detection. This leads to poor generalization performance of networks because the complex and rich contextual information around objects to be detected is often presented in real-life scenarios.

To address the above problems, it is presented in the present work that the Gate Channel Head Embedded (GCHE) module combined with the gated mechanism (GCT) and the YOLOv4 predictive network.

**Figure 11:**
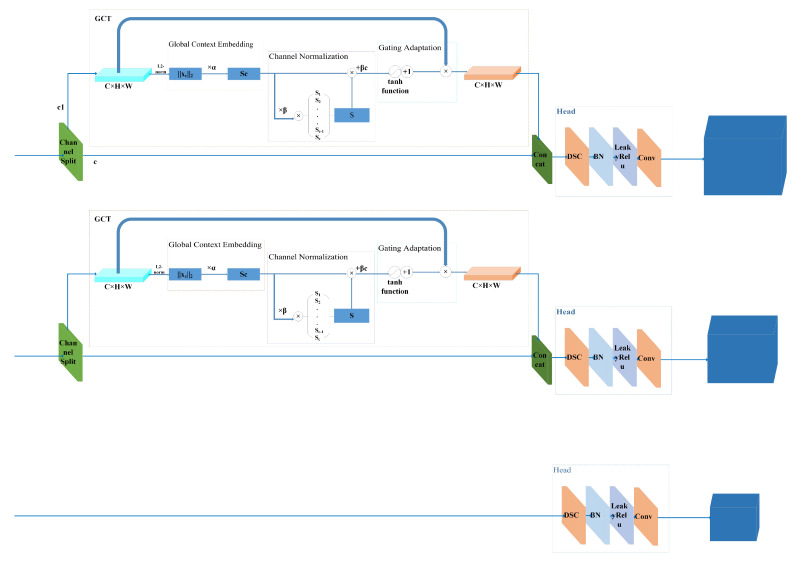
GCHE module structure.

As shown in Figure 11, the input feature information first undergoes a random channel separation operation, and the feature channel is divided into two parts c and c1. c1 re-weights the picture information through the GCT module, and finally superimposes the acquired feature information with c, and inputs it into the head network for feature extraction. In this paper, the GCHE module is embedded into the SCUPA module, as shown in Figure 4, and the feature reuse mechanism of the SCUPA module is used to further strengthen the prediction network’s ability to extract image features information.

### 3.5. Efficient Complete Intersection over Union (ECIoU) Regression Loss Function

Because direct lightweight processing of target detection algorithm networks may lead to a decrease in detection performance, more and more scholars considered improving the part of the algorithm except for the model structure without increasing the network complexity. Because the target detection can be generally divided into two parts: positioning and detection, where the accuracy of positioning is mainly dominated by the regression loss function. Therefore, many new regression loss functions have been proposed [35,36,37].

By selecting the appropriate positive and negative samples, the Intersection over Union (IoU) plays the most popular indicator of the boundary box regression, whose function is to measure the similarity between the prediction box and the real box. To further obtain the optimal IoU metric, the IoU loss function [38] is proposed to improve the IoU metrics. However, the IoU loss function does not work when the prediction box and the IoU loss function do not overlap. To solve these problems, many different evaluation systems are derived based on IoU, which improves the defects existing in the original IoU loss function from different aspects and greatly enhances its robustness. The most representative methods are Generalized Intersection over Union (GIoU) [35], Distance Intersection over Union (DIoU) [36], and (Complete Intersection over Union) CIoU [36] loss functions, which play fundamental roles in great advances in target detection, but still has a great space for optimization.

In the above method, CIoU is currently the best-performing border regression loss function, which puts three important geometric factors into consideration: the overlap area, center point distance, and aspect ratio. The CIoU measures the overlap area of the target and real boxes with the IoU, the Euclidean distance, and the corresponding aspect ratio with the angle. The loss function is as follows.

(10) $$L_{CIoU}=1-IoU+\frac{\rho^{2}\left(b,b^{gt}\right)}{c^{2}}+\alpha v,$$

(11) $$\alpha =\frac{v}{1-IoU+v},$$

(12) $$v=\frac{4}{\pi^{2}}{\left(arctan\frac{w^{gt}}{h^{gt}}-arctan\frac{w}{h}\right)}^{2},$$

where, $\rho (b,b^{gt})$ represents the Euclidean distance at the center point of the prediction box and the real box, *c* represents the diagonal distance at the minimal closure region capable of containing both the prediction box and the real box, α represents the weight coefficient, *v* measures the consistency between the aspect ratio between the prediction box and the true box. $w^{gt}$ is the width of the real box, $h^{gt}$ is the height of the real box, *w* is the width of the prediction box and *h* is the height of the prediction box.

However, the last term *v* of the CIoU loss function uses the arctangent function to form the penalty term of the rectangular aspect ratio with the following two problems, which affect the convergence speed and robustness of CIoU.

1. The *v* robustness is weak, sensitive to the outliers, and is greatly affected by the outliers, resulting in large fluctuations in the value change of the loss function, and affecting the performance of the loss function.

2. The value domain of the arctangent function is (0,π/2), which cannot directly meet the normalization requirements required by the loss function. New coefficients are introduced to realize the numerical normalization of the penalty term, and add computational complexity.

To solve the above problems, we propose a more efficient and direct regression loss function ECIoU based on the CIoU loss, which is defined as follows.

(13) $$L_{ECIoU}=1-IoU+\frac{\rho^{2}\left(b,b^{gt}\right)}{c^{2}}+\beta \theta ,$$

(14) $$\beta =\frac{\theta }{1-IoU+\theta },$$

(15) $$\theta ={\left(\frac{1}{1+e^{-\frac{w^{gt}}{h^{gt}}}}-\frac{1}{1+e^{-\frac{w}{h}}}\right)}^{2}.$$

The last term θ of the ECIoU loss function presented in this paper abstracts the rectangular aspect ratio as the definition domain of the Sigmoid (y=1/(1+exp(−x))) function, abstracts the difference between the prediction box and the real box aspect ratio as the value domain of the function, and optimizes the loss function penalty term using the function idea. The penalty term θ is more robust and smooth than the CIoU loss function the penalty term *v*, and its function output value is (0,0.25), which can directly meet the requirements of normalization, simplify the complexity of the original loss function, so that the regression loss function ECIoU can obtain faster convergence rate, better positioning results and model performance.

**Figure 12:**
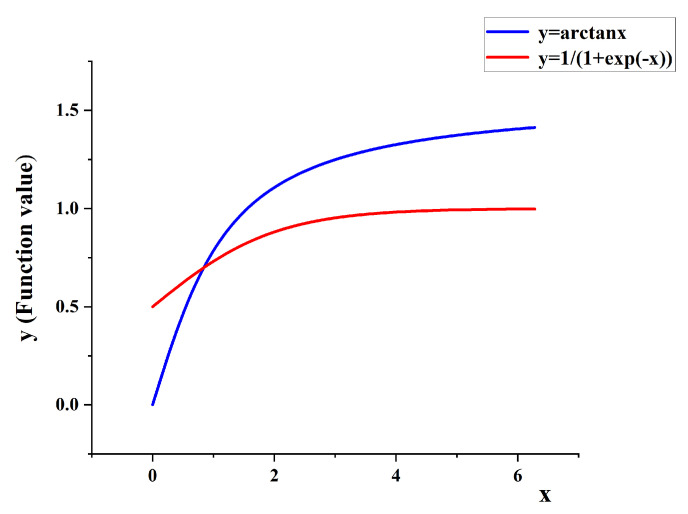
Comparison chart of different functions.

It can be clearly seen from Figure 12 that the Sigmoid function is smoother than the arctangent function, and the variation range of the function value is more stable.

**Figure 13:**
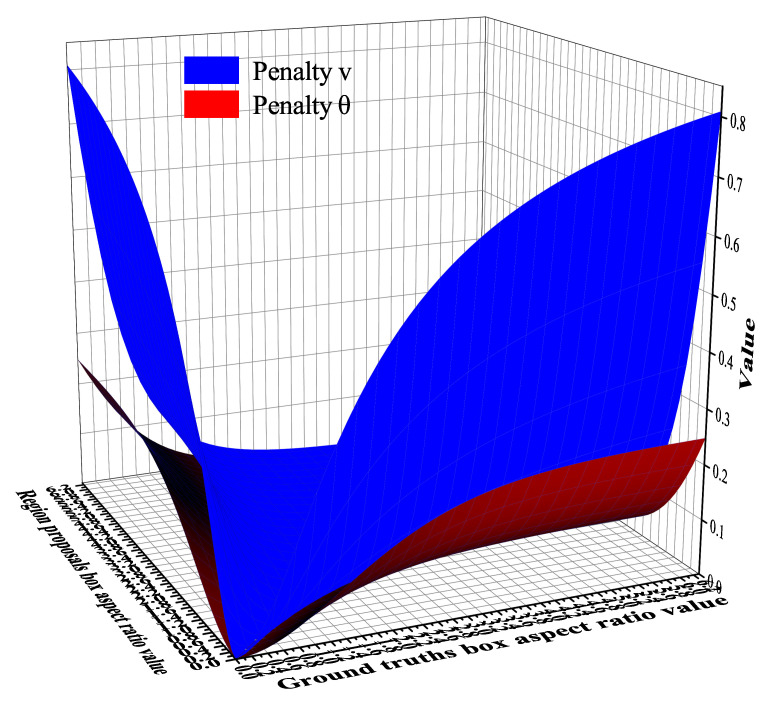
Experimental simulation diagram.

To further explore the applicability of the penalty term θ, we randomly select a part of the values as the border aspect ratio to simulate the numerical change curves of the different penalty terms of the regression loss function during the training process. Figure 13 shows the experimental simulation diagram of the penalty term in the regression loss function. We can see that the penalty term presented in this paper has a more gentle gradient, more robust for anomalies, smaller regression error and better regression effect than the original penalty term of the CIoU loss function.

## 4. Experiments

This section demonstrates the performance of Efficient-YOLO through some experiments.

### 4.1. Dataset and Experimental Conditions

We use SAR Ship Detection Dataset (SSDD) [39] and High-Resolution SAR Images Dataset (HRSID) [40] datasets in our experiments to evaluate the proposed method.

The SSDD is the first publicly available satellite remote sensing dataset, specifically used for SAR image ships by using the PASCAL_VOC annotation format. Images were mainly obtained from the RadarSat-2, TerraSAR-X, and Sentinel-1 sensors, and were obtained by using HH (horizontal transmit/horizontal receive), HV (horizontal transmit/vertical receive), VV (vertical transmit/vertical receive), and VH (vertical transmit/horizontal receive) polarization methods. The dataset is divided into ship targets for many different scenarios, including simple scenarios with a clean background, complex scenarios with many disturbances, and scenes close to the shore. The SSDD has 1160 images, and 2456 ships, with an average of 2.12 ships per image. We randomly selected 930 as the training set and the remaining 230 as the test set.

HRSID is a large-scale ship detection dataset with 5604 images of size 800 × 800 and 16,951 ships. The SAR ship imagery in HRSID comes from Sentinel-1 and TerraSAR-X and is capable of 0.1 m to 3 m resolution. Compared with SSDD, the HRSID dataset has higher resolution, more complex scenes and richer ship feature information. We divide the entire dataset into two parts: training set (4034) and test set (1570).

To ensure the consistency of the comparative experimental environment and enhance the credibility of the experimental results, all the experimental results in this paper are based on the same dataset and run on the same device.

The software and hardware platform parameters implemented by the algorithm in this paper are shown in Table 1.

### 4.2. Experimental Evaluation Metrics

To evaluate the performance of the model and demonstrate the effectiveness of the proposed Efficient-YOLO, the following metrics are selected for performance evaluation.

As the measure of detection accuracy in object detection, the formula for Mean Average Precision (mAP) is

(16) $$mAP=\frac{1}{N}\sum_{i=1}^{N}AP\left(i\right),$$

where *N* represents the number of all categories, *AP* is the mean of accuracy at different recall rates used to evaluate the detection accuracy of a certain class of samples in the dataset, here we give the formula.

(17) $$AP=\int_{0}^{1}P\left(R\right)dR,$$

where *P* is the accuracy of a certain class of samples refers to the ratio of the number of positive samples detected by the network model to the number of all samples detected. *R* represents recall which means the probability of all the number of positive samples correctly detected by the network, and the accuracy *P* and recall *R* are formulated as follows.

(18) $$P=\frac{TP}{TP+FP},$$

(19) $$R=\frac{TP}{TR+FN},$$

where *TP* represents the number of samples correctly divided into positive, namely the target categories detected consistent with the true ones. *FP* is the number of samples predicted to be positive but actually negative and *FN* is the number of samples predicted to be negative but actually positive.

As a measure of the classification problem, *F*1 score usually serves as the final measure of the multi-classification problem which is the harmonic average of precision and recall. The larger the *F*1 value, the better the model performance. The *F*1 scores in individual categories can be calculated as

(20) $$F1=\frac{2*P*R}{P+R}.$$

Frames Per Second (FPS) means the number of pictures that can be detected in the object detection network per second. FPS is referred as the index which is used to evaluate the detection speed of the object detection network. A larger FPS implies a faster network detection speed.

The Log-Average Miss Rate (LAMR) indicates the omission of the test set in the dataset. Larger LAMR indicates more missed targets; smaller LAMR implies and less missed targets and better model performance.

FLOPs is short for floating point of operations, the number of floating-point operations that can be used to measure algorithm or model complexity. The smaller the FLOPs, the lower the computational complexity of the network, the better the effect.

### 4.3. Comparison of Different Methods Based on SSDD Dataset

In order to demonstrate the effectiveness of the Efficient-YOLO algorithm in the present paper, several different object detection algorithms were selected based on the same dataset and the same device, comparing various performance indicators and verifying the algorithm performance under various complex conditions to detect the performance of the algorithm.

As can be learned from Table 2, compared with other state-of-the-art methods, the method proposed in this paper, Efficient-YOLO, has achieved amazing improvements in various performance metrics. In particular, amazing improvements are achieved in the lightweight of network models, such as the number of network parameters and model size of Efficient-YOLO is only one-eighth that of YOLOv4, the computational complexity is only one-third that of SSD, and the detection speed is more than twice that of YOLOv4. Although the network model of Efficient-YOLO is very thin, its detection performance improves to different degrees rather than decreases compared to other methods. For example, the mAP of Efficient-YOLO is 32.03% higher than that of RetinaNet, P is 5.16% higher than that of YOLOX-L, and R is 18.9% higher than that of CenterNet. The comparison of the above data indicators proves the scientificity and applicability of the target detection algorithm proposed in this paper, and achieves a good balance between detection accuracy and detection speed, which not only ensures the simplicity of the network model architecture, greatly improves the detection speed, but also ensures the excellent detection performance.

**Figure 14:**
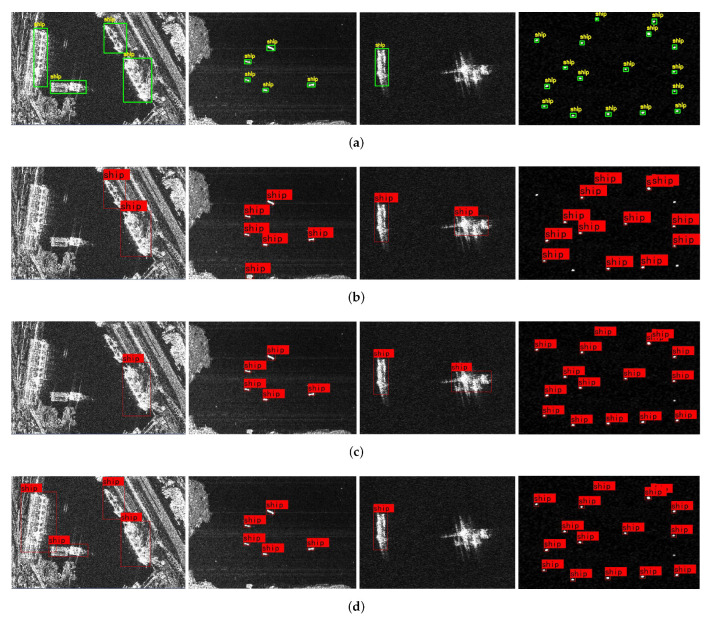
Visual analysis of the algorithm performance. (**a**) The visualization of the SSDD dataset labels; (**b**) SSD detection results; (**c**) YOLOv4 detection results; (**d**) Efficient-YOLO detection results.

It can be clearly seen from Figure 14 that the Efficient-YOLO algorithm proposed in this paper has a good application effect for the small scale, dense arrangement and error-prone targets in complex scenarios, which greatly reduces the occurrence of missed and false detection. For example, in the first figure of Figure 14a, it is not difficult to find that the ship’s target characteristics are weak and integrated into the surrounding environment. This situation leads to serious leak detection problems in other algorithms, while Efficient-YOLO can correctly detect all the targets in the picture, indicating that the algorithm has extremely strong feature extraction capability and detection performance. In the third picture of Figure 14a, both SSD and YOLOv4 have the false inspection, identifying non-ship targets with highly similar image characteristics as ship targets, while Efficient-YOLO can well block the influence of the interference targets and accurately detect the correct targets, indicating that Efficient-YOLO is highly robust to remote sensing ship targets.

**Figure 15:**
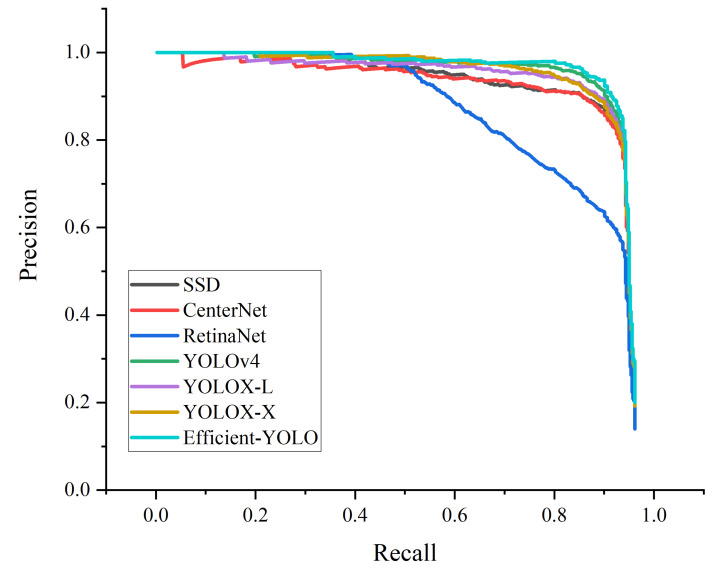
The PR curves of the different target detection methods on the SSDD datasets.

Figure 15 shows the PR curve. The area enclosed by the PR curve and the coordinate axis is the mAP value. It can be clearly seen that the mAP value of Efficient-YOLO is higher than other advanced algorithms.

### 4.4. Ablation Experiment

In this section, different combinations of modules were set up to analyze the effects of the ECIoU regression loss function, SCUPA and GCHE modules on the performance of the algorithm separately. The benchmark network of this experiment is Orignal-YOLO, which takes Mobilenetv3 as the main stem extraction network, PANet and SPP are the neck network, the prediction network of YOLOv4 is the prediction network and the regression loss function is CIoU. The SSDD dataset was selected for the experiment, with an input image size of 512 × 512, a batch size of 4, and a training step of 100 epochs. The model hyperparameter initialization is shown in Table 3.

As shown in Table 4, when different modules are combined together, the performance indicators have changed to various degrees, but not every combination of modules can bring about item-by-item performance improvements, such as the recall of the combined modules of SCUPA + GCHE compared with the individual SCUPA module, the recall rate is reduced by 2.29%, but both mAP and P are improved. The reason for this is that not each improvement technique completely independent, and some techniques are effective when used alone, but can cause some performance indicators to be ineffective when used in combination.

Therefore, here, we comprehensively consider the two aspects of network lightweight and performance efficiency, and give an incremental way of the network module combination order with the best overall effect: ECIOU loss function + SCUPA + GCHE. It is clearly seen from Table 4 that the proposed method combination dramatically improves the network performance based on the same benchmark network. Although P decreased slightly compared to Orignal-YOLO, its recall rate increased by 16.88%, LAMR decreased by 8%, mAP increased by 4.6%, and the model size and FLOPs were only 72.68% and 71.21% of Orignal-YOLO.

**Figure 16:**
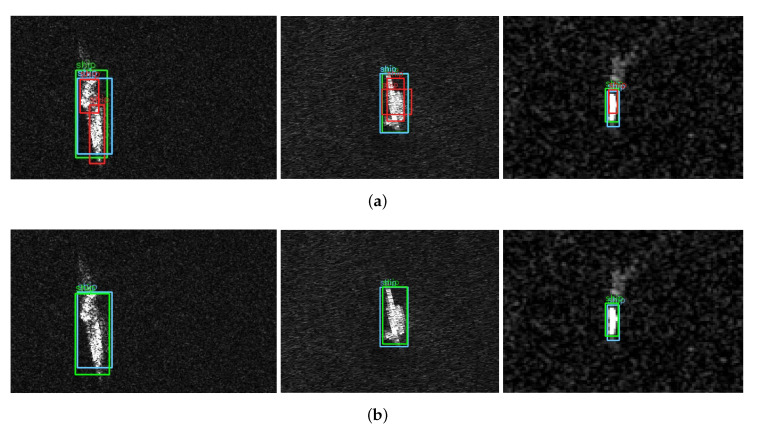
Single target visualization comparison between Original-YOLO and Efficient-YOLO. Blue boxes indicate ground truth. Green boxes and red boxes indicate prediction boxes, green boxes are positive samples, and red boxes are negative samples. (**a**) Original-YOLO; (**b**) Efficient-YOLO.

**Figure 17:**
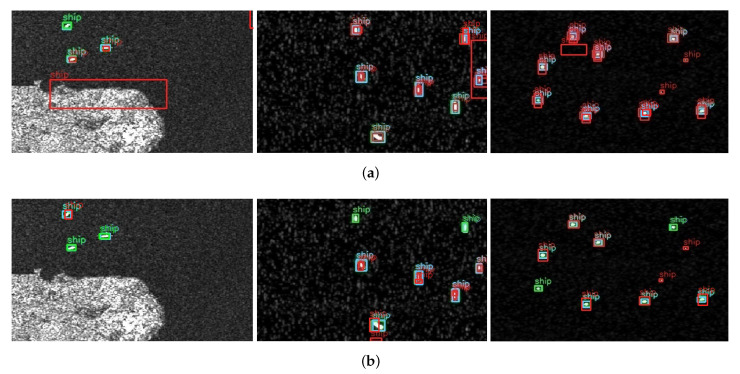
Comparison of dense target visualization between Original-YOLO and Efficient-YOLO in complex situations. Blue boxes indicate ground truth. Green boxes and red boxes indicate prediction boxes, green boxes are positive samples, and red boxes are negative samples. (**a**) Original-YOLO; (**b**) Efficient-YOLO.

It can be clearly seen from Figure 16 that Efficient-YOLO has a good application effect on a single ship target. The prediction box of the network is highly coincident with the target real box, and each ship target can be accurately detected, and no negative sample prediction box appears. It shows that Efficient-YOLO has strong precision and robustness. To further explore the applicability of the network for dense ship targets in complex situations, we perform experiments on Original-YOLO and Efficient-YOLO separately, yielding multiple visual images in Figure 17. From this analysis, we can find that Efficient-YOLO still has a good detection effect even in complex cases, which can greatly reduce the appearance of a negative sample prediction box and effectively improve the detection performance of the network. The results show that the proposed ECIOU loss function, SCUPA and the GCHE module can greatly enhance the representation ability of feature maps and suppress interference information.

### 4.5. Experimental Analysis of the ECIoU Regression Loss Function

In this section, we replace the ECIoU regression loss function in Efficient-YOLO with the DIoU and CIoU regression loss functions, respectively. Taking the performance of the DIoU loss function as the benchmark performance, the effectiveness of the ECIoU regression loss function proposed in this paper is analyzed in a variety of complex situations.

As shown in Table 5, the ECIoU regression loss function presented in this paper has a better performance on each metric compared to the DIoU and CIoU loss functions. For example, the ECIoU loss function achieved 3.46%, 10.14% and 5.81% gain on mAP, R and F1, and the LAMR decreased by 34.78%, achieving an amazing performance improvement over the CIoU loss function performance, thus confirming the effectiveness of the ECIoU loss function. Although the accuracy of ECIoU loss function is slightly lower compared to CIoU loss function, the slight reduction in accuracy compared with the huge improvement of other performance indicators is negligible. Because of the target detection algorithm in practice, the most important performance evaluation index or the average accuracy, recall rate and log average missed detection rate.

**Figure 18:**
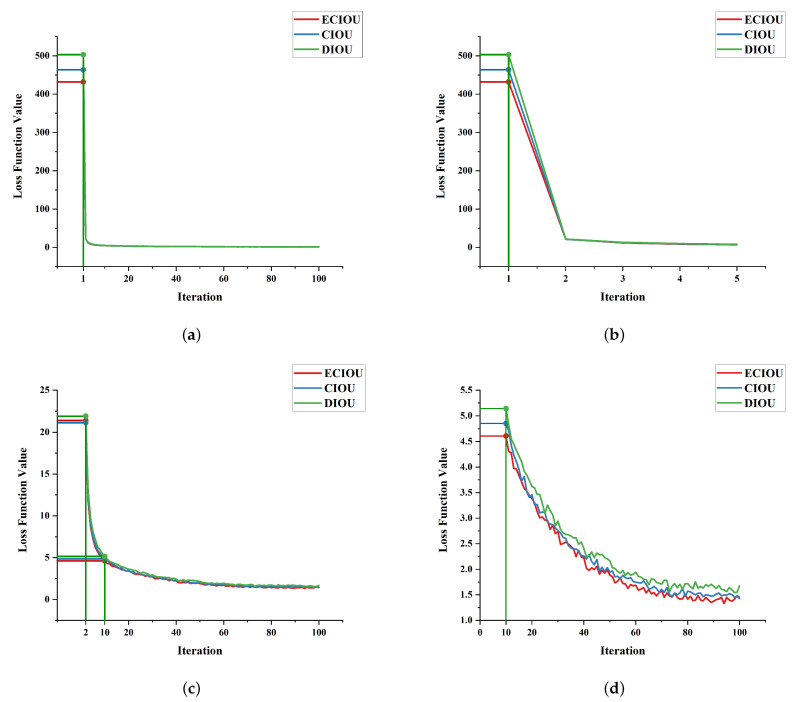
The experimental regression loss functions for iterations: (**a**) from 1 to 100; (**b**) from 1 to 5; (**c**) from 2 to 100; (**d**) from 10 to 100.

Figure 18a is the original picture of the comparison of loss function values. Due to the dense experimental data, it is difficult to clearly distinguish the performance of each loss function in this comparison chart. Therefore, Figure 18a is decomposed into Figure 18b–d to analyze the experimental results. Figure 18b is obtained by partially enlarging the value of the initial iteration loss function of each loss function in Figure 18a. Since the model is being built for the first time, there are no pretrained weights. When training starts, the value of the first iteration is relatively large, and after the second iteration, the value of the loss function quickly converges within 25. The difference between the values before and after is large, and it is difficult to compare the decline curves of different loss functions. Therefore, the first iteration value is temporarily removed, resulting in a loss function Figure 18c from 2 to 100 epochs. Figure 18c is partially enlarged to obtain Figure 18d.

It can be clearly seen from Figure 18b–d that, compared with other loss functions, after using the ECIoU loss function, the value of the first iteration loss function of the target detection algorithm model is smaller, and during the whole experiment process, the loss function value drops faster, and the function value is generally smaller than other loss functions, indicating that the ECIoU loss function has faster convergence speed and better generalization performance.

**Figure 19:**
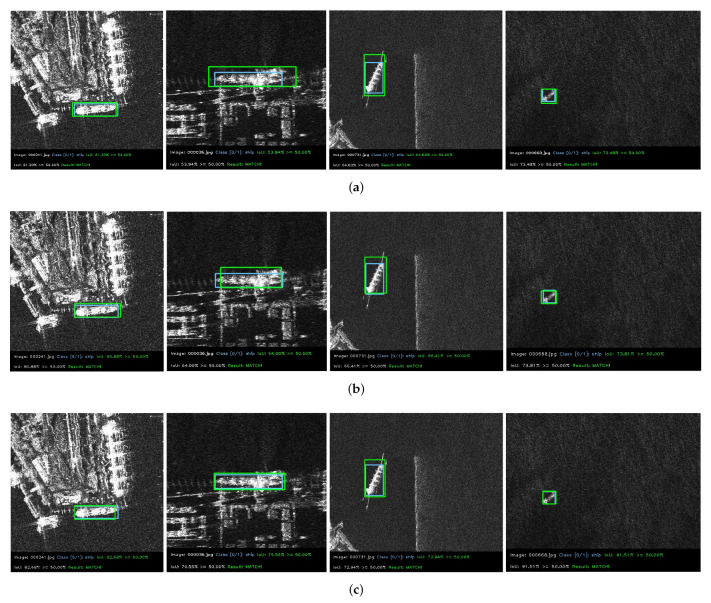
Visualization of localization matching with different loss functions. Blue boxes indicate ground truth. Green boxes indicate prediction boxes. (**a**) DIoU; (**b**) CIoU; (**c**) ECIoU.

In a variety of complex scenarios, comparing the CIoU and DIoU loss functions, we can clearly find from Figure 19 that the localization ability of the ECIoU loss function has been significantly improved. The predicted box and the target real box are highly matched and can accurately locate the target to be detected. It shows that the ECIoU loss function has strong stability and applicability to effectively distinguish complex backgrounds and improve network performance.

### 4.6. Detection Results on the HRSID Dataset

In order to further test the generalization performance of Efficient-YOLO, we select the HRSID dataset with a larger scale and more complex environment to detect the performance effects of input image sizes of 640 × 640 and 800 × 800, respectively.

As shown in Table 6, compared with other advanced object detection networks, the detection performance of the network proposed in this paper is still excellent on large-scale datasets, and the performance indicators are excellent. This shows that Efficient-YOLO has good generalization performance, not only limited to a single dataset, but also has the potential to be popularized on more complex datasets.

**Figure 20:**
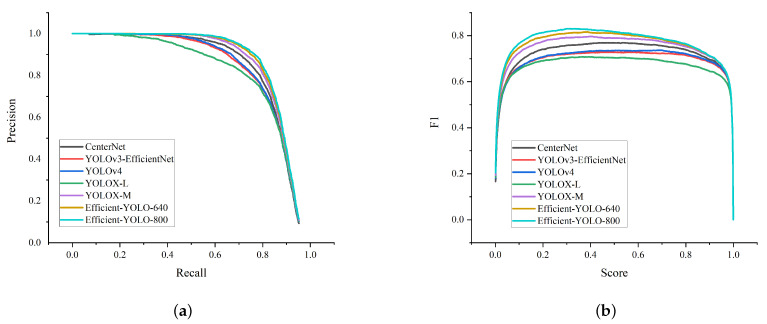
Comparison of different target detection performance. (**a**) PR curves; (**b**) F1 curves.

To further explore the Efficient-YOLO network, we additionally select the HRSID dataset and the new network for comparative experiments. From Figure 20, we can see that Efficient-YOLO can still maintain better detection ability than other state-of-the-art networks even on more difficult-to-detect datasets. It is clear from the figure that the PR and F1 curves of Efficient-YOLO are steadily higher than the other methods from the beginning to the end, and the curve does not show a sharp drop. It shows that the Efficient-YOLO algorithm has excellent target feature information transmission ability, excellent feature information acquisition ability and excellent image feature positioning information access ability, so that the algorithm has ideal detection performance.

**Figure 21:**
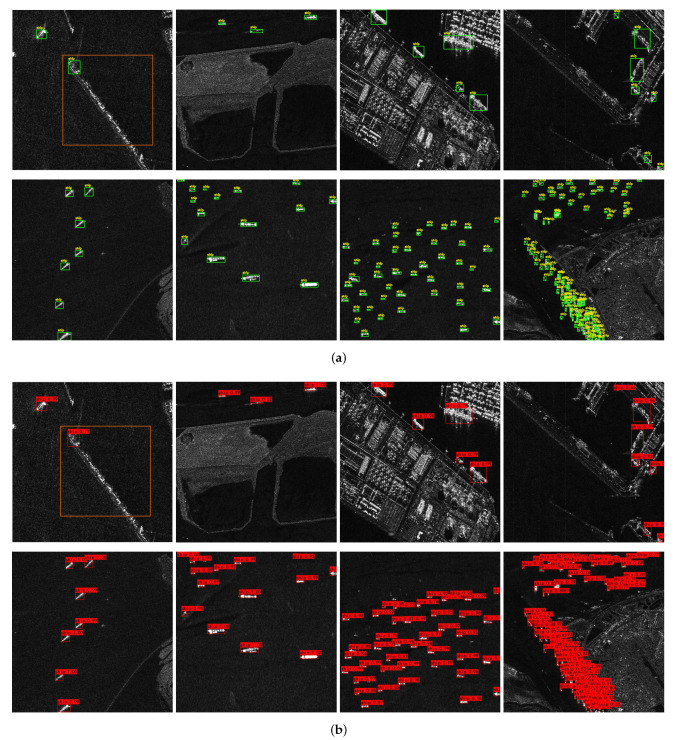
Visualize detection results. (**a**) The visualization of the HRSID dataset labels; (**b**) Efficient-YOLO detection results.

As shown in Figure 21, we select images of ships with ocean waves, near-shore building disturbances and dense targets for detection, respectively, to evaluate network performance. In the orange box in the figure, the target of the ship is confused with the waves generated during the journey. It is difficult for us to distinguish the position of waves and ships with naked eyes, but Efficient-YOLO can accurately detect the corresponding ship targets. The detection effect is also ideal when the network is applied to other dense ship targets. From this, we can find that Efficient-YOLO has strong interference information suppression ability and ideal target detection ability, and can accurately detect ship targets.

## 5. Discussion

Most current object detection algorithms mainly focus on a single aspect of detection accuracy and network real-time performance, and few methods can achieve a balance between them. For example, some scholars will continue to expand the network structure in order to obtain the ideal detection effect, resulting in poor network real-time performance, making it difficult to apply related algorithms to practice.

This paper presents a novel and efficient lightweight network, Efficient-YOLO, for SAR ship target detection in complex environments. The network can quickly locate the ship’s target area, significantly reducing the computational amount and missed detection rate of ship detection. The SCUPA module in the network structure can effectively improve the transmission ability of the image feature information, ensure the integrity of the target information to be detected, and further reduce the false detection rate of the ship target detection. GCHE module can minimize the impact of interference information on image information, ensure as much as possible that the network obtains the target, and enhance the target detection capability of the network. The ECIoU loss function has a faster convergence speed and robustness, which can help the model achieve the ideal detection effect at the fastest speed in complex environments.

Looking at the comparison of the above performance indicators and visualizations, it can be clearly seen that the performance of the Efficient-YOLO network is superior and can easily cope with various complex environments. This shows that the improved method proposed in this paper is effective and the cited architectural patterns are reasonable. However, we also noticed from Table 2 and Table 6 that although the logarithmic missed detection rate of Efficient-YOLO is very low, its missed detection rate still exists. We will continue to explore corresponding solutions in future research, such as: improving the GCHE module with a decoupling head structure [20].

## 6. Conclusions

The research carried out in this paper introduces an efficient and lightweight SAR image marine environment ship detection method based on deep learning. According to the above experimental results of ship detection in complex scenarios, this method achieves an ideal balance between accuracy and speed with a simplified network structure, better regression loss function, SCUPA and GCHE module. The overall effect is better than other advanced object detection algorithms. Summarized as follows.

1. A new regression loss function ECIOU is proposed. Its formula is simpler, the matching degree between the detection boxes and the ground truth is higher, the convergence speed is faster, and the application effect is better.

2. We propose the SCUPA module, while reducing the computational complexity of the network, it can strengthen the multiplexing of image feature information, and effectively improve the generalization performance and detection performance of the model.

3. The GCHE module is proposed to effectively shield the interference information of the target to be detected and enhance the ability of the network to extract feature information.

The network model Efficient-YOLO proposed in this paper has strong real-time performance, small model size and high detection accuracy, making it a broader application prospect in the field of ocean monitoring. Efficient-YOLO only performs experiments on SSDD and HRSID datasets in this paper, and in the future we will focus on performing performance validation on more ship datasets. 

## Figures and Tables

**Table 1:** Experimental platform environment configuration.

Name	Configuration
CPU	Intel (R) Core (TM)i7-11700K @ 3.60GHz
GPU	NVIDIA GeForce RTX 3060 12 GB
Operating system	Window 10
Deep learning framework	Pytorch 1.9.0
Programming language	Python 3.7
Dependent package	CUDA 11.1 + CUDNN 8.0.4

**Table 2:** Experimental platform environment configuration.

Method	SSD	CenterNet	RetinaNet	YOLOv4	YOLOX-L	YOLOX-X	Efficient-YOLO
Backbone	VGG-16	ResNet-50	ResNet-50	CSPDarknet-53	Modified CSP v5	Modified CSP v5	MobileNetv3
Size	512	512	512	512	512	512	512
Parameters	2.36 × 10${}^{7}$	3.27 × 10${}^{7}$	3.63 × 10${}^{7}$	6.39 × 10${}^{7}$	5.41 × 10${}^{7}$	9.90 × 10${}^{7}$	0.82 × 10${}^{7}$
Model Size	90.07 MB	124.61 MB	138.59 MB	243.90 MB	206.56 MB	377.64 MB	31.34 MB
LAMR	27	32	44	20	25	23	15
mAP	89.55	83.31	63.71	91.17	88.66	88.92	93.56
P	91.30	93.57	96.61	95.04	90.93	90.48	96.09
R	73.21	66.79	52.29	84.40	84.59	83.67	85.69
F1	81	78	68	89	88	87	91
FPS	42.71	59.70	33.33	31.35	36.73	19.16	68.52
FLOPs	87.59G	22.15G	26.01G	45.26G	49.7G	90.08G	3.81G

**Table 3:** Initialization of the model hyperparameters.

Hyperparameter	Initialization
Learning rate	0.001
Image size	512
Batch size	4
Train epoch	100
Weight decay	0.0005
Momentum	0.937
Label smoothing	0.005

**Table 4:** Experimental platform environment configuration.

ECIoU	SCUPA	GCHE	Model Size	mAP	P	R	F1	LAMR	FLOPs
			43.12 MB	88.96	97.40	68.81	81	23	5.35G
✓			43.12 MB	90.82	96.88	73.94	84	21	5.35G
	✓		31.34 MB	92.09	95.42	85.04	89	19	3.81G
		✓	43.12 MB	91.04	95.64	80.55	87	21	5.35G
✓	✓		31.34 MB	93.56	96.99	82.75	89	16	3.81G
✓		✓	43.12 MB	90.82	96.04	80.00	87	22	5.35G
	✓	✓	31.34 MB	92.49	96.37	82.75	89	19	3.81G
✓	✓	✓	31.34 MB	93.56	96.09	85.69	91	15	3.81G

**Table 5:** Quantitative analysis using different regression loss functions (larger mAP, P, R, F1, smaller LAMR, representing better performance).

Loss/Evaluation	mAP	P	R	F1	LAMR
DIoU	90.43	95.28	77.80	86	23
CIoU	92.49	96.37	82.57	89	19
Relative improve.%	2.28%	1.14%	6.13%	3.49%	−17.39%
ECIoU	93.56	96.09	85.69	91	15
Relative improve.%	3.46%	0.85%	10.14%	5.81%	−34.78%

**Table 6:** Comparison of performance indicators of different algorithms.

Method	CenterNet	YOLOv4	YOLOv3-EfficientNet	YOLOX-L	YOLOX-M	Efficient-YOLO	Efficient-YOLO
Backbone	ResNet-50	CSPDarknet-53	Efficient-B2	Modified CSP v5	Modified CSP v5	MobileNetv3	MobileNetv3
Size	640	640	800	800	800	640	800
Parameters	3.27 × 10${}^{7}$	6.39 × 10${}^{7}$	1.56 × 10${}^{7}$	5.41 × 10${}^{7}$	2.53 × 10${}^{7}$	0.82 × 10${}^{7}$	0.82 × 10${}^{7}$
Model Size	124.61 MB	243.90 MB	59.48 MB	206.56 MB	96.44 MB	31.34 MB	31.34 MB
LAMR	40	43	44	52	34	31	29
mAP	73.99	73.98	75.05	71.98	81.80	85.06	87.06
P	96.06	92.72	89.74	84.02	94.20	92.24	93.36
R	57.50	59.06	60.49	59.91	66.89	72.16	72.54
F1	72	72	72	70	78	81	82
FPS	41.61	32.47	29.13	18.33	28.55	66.14	49.67

## Data Availability

The data presented in this study are available on request from the corresponding author.
